# Host-Associated Absence of Human Puumala Virus Infections in Northern and Eastern Germany

**DOI:** 10.3201/eid2301.160224

**Published:** 2017-01

**Authors:** Stephan Drewes, Hanan Sheikh Ali, Moritz Saxenhofer, Ulrike M. Rosenfeld, Florian Binder, Fabian Cuypers, Mathias Schlegel, Susanne Röhrs, Gerald Heckel, Rainer G. Ulrich

**Affiliations:** Friedrich-Loeffler-Institut, Greifswald-Insel Riems, Germany (S. Drewes, H. Sheikh Ali, U.M. Rosenfeld, F. Binder, F. Cuypers, M. Schlegel, S. Röhrs, R.G. Ulrich);; Sudan University of Science and Technology, Khartoum, Sudan (H. Sheikh Ali);; University of Bern, Bern, Switzerland (M. Saxenhofer, G. Heckel);; Swiss Institute of Bioinformatics, Lausanne, Switzerland (M. Saxenhofer, G. Heckel)

**Keywords:** bank vole, evolutionary lineage, phylogroup, *cytochrome b*, Puumala virus, nephropathia epidemica, outbreak, endemic region, Germany, Central Europe, zoonoses, viruses

## Abstract

Human hantavirus disease cases, caused by Puumala virus (PUUV), are mainly recorded in western and southern areas of Germany. This bank vole reservoir survey confirmed PUUV presence in these regions but its absence in northern and eastern regions. PUUV occurrence is associated with the presence of the Western bank vole phylogroup.

Puumala virus (PUUV) causes most hantavirus disease cases in Central and Western Europe and is the only human pathogenic hantavirus in Fennoscandia (*1*). The human infection is characterized by a mild-to-moderate form of hemorrhagic fever with renal syndrome designated nephropathia epidemica (NE), with a case fatality rate of <0.1%. The only virus reservoir in Central and Western Europe is the bank vole, *Myodes glareolus* (*1*).

PUUV causes most human hantavirus infections in Germany, with an incidence of 10.31 cases/100,000 inhabitants (*2*). Human disease reports fluctuate temporally with peaks in the years 2007, 2010, and 2012, but reports also show a heterogeneous spatial distribution (*2*,*3*). Generally and during outbreak years, the highest numbers of cases occurred in the western and southern parts of Germany, whereas in the northern and eastern parts of the country only a few cases were recorded (Figure 1, panel A).

**Figure 1:**
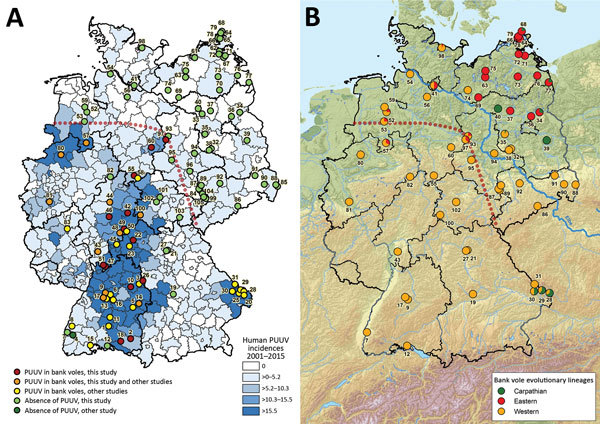
Geographic distribution of Puumala virus (PUUV)–positive and PUUV-negative bank voles in Germany (A) and assignment of bank voles to the evolutionary lineages Western, Eastern, and Carpathian (B). The coloration of the map in panel A was generated on the basis of the human PUUV incidence per district (*2*). PUUV detection in previous studies was extracted from (*3*–*7*). The identification of the bank vole evolutionary lineages shown in panel B was determined by using partial *cytochrome* *b* gene sequences (see Figure 2). The red dotted line illustrates the hypothetical current edge of the range of PUUV-positive bank voles.

Molecular analyses of bank voles from endemic regions detected the presence of PUUV at 30 sites in Germany (Figure 1, panel A) and resulted in the definition of several PUUV sublineages of the Central European (CE) clade (*3*,*8*). In addition, an 8-year monitoring study on the bank vole populations in a PUUV-endemic region of northwestern Germany indicated the long-term presence of particular PUUV strains (*4*). 

To evaluate potential reasons for the almost total absence of human PUUV infections in northern and eastern Germany, we investigated bank voles from these regions and from PUUV-endemic regions in the western and southern parts of Germany for the presence of PUUV and typed the voles to major evolutionary lineages on the basis of *cytochrome* *b* gene sequences.

## The Study

A total of 1,774 bank voles were collected by partners of the network Rodent-borne Pathogens (*3*,*5*,*6*,*9*–*11*) at sites in PUUV-endemic regions of western and southern Germany and sites in the eastern and northern parts of Germany (Figure 1, panel A; Technical Appendix Table). Chest cavity lavage samples of voles were investigated by IgG ELISA using a recombinant nucleocapsid protein of PUUV (*6*). For molecular PUUV detection, RNA was isolated from lung or heart tissue by using a QIAzol Lysis Reagent (QIAGEN, Hilden, Germany) extraction protocol. The RNA samples were subjected to small (S) segment reverse transcription PCR (RT-PCR) with primer pair Pu342F and Pu1102R (6), and the resulting cDNAs were sequenced. RNA samples were also subjected to a novel PUUV S segment–specific real-time RT-PCR with primers PUUV S-broad-F (5′-AACCCGCCATGAACAACAAC-3′) and PUUV S-broad-R (5′-TGCTGACACTGTTTGTTGCC-3′) and fluorescence reporter probe PUUV S-broad (5′ 6-FAM-GGAAATGGACCCAGATGACGT-BHQ-1 3′) (for further details see footnote of Technical Appendix Table).

First, serologic investigation of 1,758 chest cavity lavage samples indicated 99 seropositive voles exclusively originating from the endemic regions in southern and western Germany (Figure 1, panel A; Technical Appendix Table). This analysis failed to detect any antibody-positive animals within the 1,210 bank voles of this panel originating from the eastern and northern parts of Germany. 

Subsequent conventional PUUV RT-PCR analysis of RNA samples from 440 voles (comprising 86 seropositive and 334 seronegative voles, 9 with equivocal results, and 11 not investigated because of the lack of chest cavity lavage samples) revealed 79 positive and 361 negative samples (Technical Appendix Table). All RT-PCR–positive samples again only originated from the PUUV-endemic regions. A final real-time RT-PCR investigation of 364 RNA samples, 34 being positive and 329 being negative by conventional RT-PCR, confirmed the results of the conventional RT-PCR analysis.

Including results of previously published studies (*3*,*4*,*7*), PUUV seroprevalence in the endemic regions showed an average of 23.9% and varied between 4.6% and 66.7% (Technical Appendix Table). According to the serologic and RT-PCR data, a PUUV-endemic region can be identified spanning the western and southern parts of Germany (Figure 1, panel A, below the dotted red line). In this study, the easternmost PUUV-positive sites were located in Saxony-Anhalt (site 97), Lower Saxony (site 60), and Thuringia (site 100) (*7*). The northernmost sites were located in Lower Saxony (sites 57 and 60) and Saxony-Anhalt (site 97). Nucleotide sequence determination and subsequent phylogenetic analysis showed that all PUUV sequences belong to the CE PUUV clade, which is divergent from other European PUUV lineages (Technical Appendix Figure).

To test for a potential association between PUUV distribution in the reservoir and evolutionary bank vole lineages, we isolated mitochondrial DNA from 383 selected voles by using the GeneMATRIX Tissue DNA Purification Kit (Roboklon, Potsdam, Germany) according to manufacturer’s guidelines. The *cytochrome b* PCR was performed and used for determination of the bank vole evolutionary lineages as described previously (*12*).

**Figure 2:**
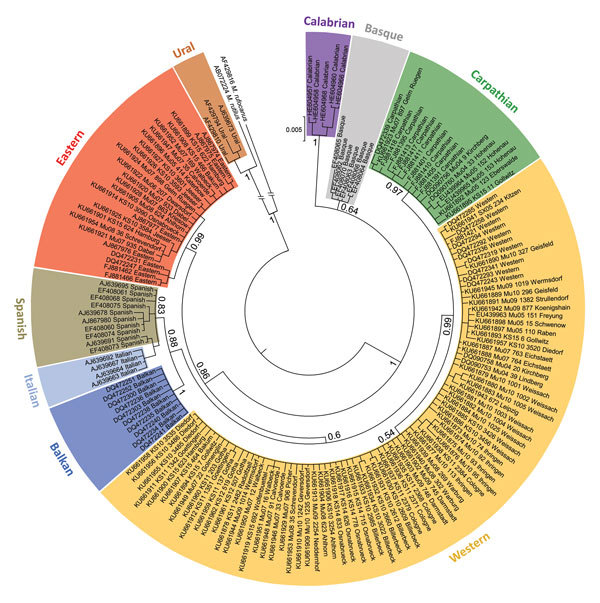
Phylogenetic relationships of European bank vole lineages. Sequences are categorized on the basis of mitochondrial *cytochrome* *b* gene sequences and shown as a maximum clade credibility phylogenetic tree with posterior probabilities displayed for major nodes. Novel sequences are labeled with individual code and trapping site (Technical Appendix Table). Additional published sequences are included as references for bank vole evolutionary lineages, labeled with GenBank accession number followed by lineage indication. Phylogenetic analyses were performed with MrBayes version 3.2.2 (https://sourceforge.net/projects/mrbayes/files/mrbayes/) on the CIPRES platform for 166 *cytochrome* *b* sequences of 843-bp length. A mixed nucleotide substitution matrix was specified in 4 independent runs of 10^7^ generations for the data set. A burn-in fraction of 25% was discarded and samples were recorded every 10^3^ generations. *Cytochrome* *b* sequences of *M. rutilus* and *M. rufocanus* voles were used as outgroups.

The *cytochrome* *b*–based typing revealed the presence of the bank vole Western, Eastern, and Carpathian evolutionary lineages (Figure 2). Most of the territory of Germany was inhabited by the Western evolutionary lineage, with its northern and eastern borders located close to the Elbe River (Figure 1, panel B). The distribution of the Eastern lineage ranged over almost the entire northern part of Germany, with partial sympatric occurrence of the Carpathian lineage in the northeast (sites 34, 68, 77) and the Western lineage in the central and northwest (sites 41, 52, 53, 57, 93, 97, 98). The Carpathian lineage was additionally located in the southeastern part of Germany (sites 28–30).

A comparison of the distribution of PUUV and the bank vole evolutionary lineages indicates an association of PUUV with the Western evolutionary lineage (Figure 1; Technical Appendix Table). This finding is in line with the detection of PUUV in Belgium and France and the exclusive occurrence of the Western evolutionary lineage in the PUUV-endemic regions of these countries (*8*,*13*,*14*). In the Bavarian Forest, the district Osnabrück (site 57), and at the easternmost distribution range in Walbeck (site 97), PUUV infections were also detected in sympatric bank voles of the Carpathian (n = 6) and Eastern (n = 7; n = 1) lineages, respectively.

## Conclusions

The occurrence of PUUV in Germany (and Belgium and France) is preferentially associated with the presence of the Western evolutionary lineage of the bank vole, but the virus was also detected in sympatric animals of the Eastern or Carpathian lineage. Future studies will have to determine if the current distribution of PUUV can be explained by the postglacial colonization of Germany by bank voles of the Western evolutionary lineage from western refugia through southern Germany (*13*–*15*).

The observed limited geographic distribution of PUUV in bank voles has important implications for public health measures and development of early warning modules for hantavirus outbreaks. These public health measures of monitoring local bank voles for PUUV strains (*4*) should be expanded to evaluate for further northeastern expansion.

Technical Appendix Serologic and molecular Puumala virus detection in bank voles from Germany and bank vole evolutionary lineage, and Puumala virus phylogenetic tree reconstructed with novel and published partial small segment sequences.
