# Supplementary material for: Host-Associated Absence of Human Puumala Virus Infections in Northern and Eastern Germany
Source: Emerg Infect Dis. 2017 Jan;23(1):83–6. doi: 10.3201/eid2301.160224 (PMC5176216; doi:10.3201/eid2301.160224)
Supplement: Technical Appendix — Serologic and molecular Puumala virus detection in bank voles from Germany and bank vole evolutionary lineage, and Puumala virus phylogenetic tree reconstructed with novel and published partial small segment sequences. [file 16-0224-Techapp-s1.pdf]

Article DOI: http://dx.doi.org/10.3201/eid2301.160224

Host-Associated Absence of Human Puumala Virus Infections in Northern and Eastern Germany

Technical Appendix

Technical Appendix Table. Serologic and molecular Puumala virus detection in bank voles from Germany and bank vole evolutionary lineage*

			Serology	S segment PCR			
Federal states of Germany	Map no.	Trapping site	IgG ELISA‡	RT-PCR§	Real-time§,¶	Reference	Bank vole phylogroup (absolute no.)
Baden-Wuerttemberg	1	Albstadt				Ettinger et al., 2012	n.d.
	2	Bad Waldsee	1/0/10	0/1	n.d.	this study	n.d.
	3	Crailsheim				Drewes et al. (in press)	n.d.
	4	Freiburg				Drewes et al. (in press)	n.d.
	5	Geislingen				Drewes et al. (in press)	n.d.
	6	Hemmingen				Ettinger et al., 2012	n.d.
	7	Ihringen	0/0/10	0/10	0/10	this study and Drewes et al. (in press)	Western (10)
	8	Kenzingen				Drewes et al. (in press)	n.d.
	9	Leonberg	0/0/5	0/5	0/5	Ettinger et al., 2012	Western (5)
	10	Michelbach-Bilz	5/0/18	3/4	n.d.	this study	n.d.
	11	Mössingen				Drewes et al. (in press)	n.d.
	12	Radolfzell	0/0/68	0/11	0/11	this study	Western (11)
	13	Renningen				Ettinger et al., 2012	n.d.
	14	Steinheim	8/0/20	5/19	n.d.	this study and Drewes et al. (in press)	n.d.
	15	Stühlingen				Drewes et al. (in press)	n.d.
	16	Stuttgart				Drewes et al. (in press)	n.d.
	17	Weissach	5/3/20[20]	7/20[20]	7/20[20]	this study, Reil, Rosenfeld et al., unpubl. data, Ettinger et al., 2012	Western (20)
	18	Zußdorf	11/1/55	7/11	n.d.	this study	n.d.
		**Total**	**30/4/206**	**22/81**	**7/46**		
Bavaria	19	Eichstätt	0/0/21	0/2	0/2	this study	Western (2)
	20	Elsenthal				Ettinger et al., 2012	n.d.
	21	Geisfeld	0/0/4	0/4	0/4	this study	Western (4)
	22	Hammelburg	1/0/5	0/1	n.d.	this study	n.d.
	23	Karlstadt				Ettinger et al., 2012	n.d.
	24	Lackenberg				Ettinger et al., 2012	n.d.
	25	Neuschönau				Schilling et al., 2007	n.d.
	26	Schnelldorf	2/0/9	0/2	n.d.	this study	n.d.
	27	Strullendorf	0/0/3	0/3	0/3	this study	Western (3)
	28	Freyung				Mertens et al., 2011	Western (1); Carpathian (2)
	29	Hohenau				Mertens et al., 2011	Western (2); Carpathian (3)
	30	Kirchberg				Mertens et al., 2011	Western (1); Carpathian (1)

Federal states of Germany	Map no.	Trapping site	Serology IgG ELISA‡	S segment PCR RT-PCR§	S segment PCR Real-time§,¶	Reference	Bank vole phylogroup (absolute no.)
	31	Lindberg				Mertens et al., 2011	Western (2)
		**Total**	**3/0/42**	**0/12**	**0/9**		
Brandenburg	32	Buchholz	†	0/1	0/1	this study	Western (1)
	33	Dreetz	0/0/8	n.d.	n.d.	this study	n.d.
	34	Eberswalde	0/0/3†	0/6†	0/7	this study	Eastern (5); Carpathian (2)
	35	Gollwitz Havel	0/0/18	0/18	0/18	this study	Western (13); Carpathian (1)
	36	Groß Schönebeck	0/0/1†	†	†	this study	Eastern (1)
	37	Katerbow	†	0/1	0/1	this study	Eastern (1)
	38	Raben	0/0/1†	†	†	this study	Western (1)
	39	Schwenow	0/0/7†	†	†	this study	Carpathian (1)
	40	Tramnitz	0/0/2	0/2	0/2	this study	Carpathian (2)
		**Total**	**0/0/40**	**0/28**	**0/29**		
Hamburg	41	Hamburg	0/0/4	0/4	0/4	this study	Western (2); Eastern (2)
		**Total**	**0/0/4**	**0/4**	**0/4**		
Hesse	42	Burghaun	2/0/7	2/2	n.d.	this study	n.d.
	43	Darmstadt	0/0/10[(2)]	0/10[(2)]	0/10	this study, Ettinger et al., 2012	Western (10)
	44	Gilserberg	2/0/7[(1)]	2/2[(1)]	n.d.	this study, Ettinger et al., 2012	n.d.
	45	Laufach				Ettinger et al., 2012	n.d.
	46	Mücke-Merlau	4/1/21	n.d.	n.d.	this study	n.d.
	47	Rothenberg	2/0/8	n.d.	n.d.	this study	n.d.
	48	Salmünster	3/0/6[(1)]	3/3[(1)]	n.d.	this study, Ettinger et al., 2012	n.d.
	49	Schlüchtern	3/0/16	3/3	n.d.	this study	n.d.
	50	Sinntal				Ettinger et al., 2012	n.d.
	51	Wald-Michelbach	1/0/22	n.d.	n.d.	this study	n.d.
		**Total**	**17/1/97**	**10/20**	**0/10**		
Lower Saxony	52	Ahlhorn	0/0/3†	0/4	0/4	this study	Western (3); Eastern (1)
	53	Bakum	0/0/12	0/12	0/12	this study	Western (9); Eastern (3)
	54	Geversdorf	0/3/15	0/14	0/10	this study	Western (10)
	55	Göttingen	4/0/26	4/13	4/13	this study	Western (13)
	56	Neddernhof	0/0/1	0/1	0/1	this study	Western (1)
	57	Osnabrück	14/2/21[(2)]	22/22[(2)]	21/21[(2)]	this study, Weber de Melo et al., 2015	Western (14); Eastern (7)
	58	Sennickerode				Ettinger et al., 2012	n.d.
	59	Wardenburg	0/0/1	0/1	0/1	this study	Western (1)
	60	Wolfenbüttel	1/1/13	0/13	0/13	this study	Western (13)
		**Total**	**19/6/92**	**26/80**	**25/75**		
Mecklenburg-Western Pomerania	61	Billenhagen	0/0/3	n.d.	n.d.	this study	n.d.
	62	Bremerhagen	0/0/7	n.d.	n.d.	this study	n.d.
	63	Dabel	0/0/5	0/2	0/2	this study	Eastern (2)
	64	Dranske	0/0/2	0/1	0/1	this study	Eastern (1)
	65	Dwarsdorf	0/0/1	0/1	0/1	this study	Eastern (1)
	66	Trent Rügen	0/0/1	0/1	0/1	this study	Eastern (1)
	67	Frätow	0/0/1	n.d.	n.d.	this study	n.d.
	68	Gelm Rügen	0/0/4†	0/5	0/5	this study	Eastern (4); Carpathian (1)
	69	Grabow	0/0/4	0/1	0/1	this study	Eastern (1)
	70	Hohenzieritz	0/0/1	n.d.	n.d.	this study	n.d.
	71	Island of Riems	0/0/1	0/1	0/1	this study	Eastern (1)
	72	Jeeser	0/0/264[(210)]	0/44[(43)]	0/44[(43)]	this study, Reil, Rosenfeld et al., unpubl. data	Eastern (44)

			Serology	S segment PCR			
Federal states of Germany	Map no.	Trapping site	IgG ELISA‡	RT-PCR§	Real-time§,¶	Reference	Bank vole phylogroup (absolute no.)
	73	Kriesow	0/0/72	0/1	0/1	this study	Eastern (1)
	74	Picher	0/0/77	0/1	0/1	this study	Western (1)
	75	Penzin	0/0/24	0/1	0/1	this study	Eastern (1)
	76	Rothemühl	0/0/32	0/4	0/4	this study	Eastern (4)
	77	Rothenklempenow	0/0/93	0/27	0/27	this study, Morger et al., 2015	Eastern (21); Carpathian (6)
	78	Seehof	0/0/3	0/2	0/2	this study	Eastern(2)
	79	Wittower Fähre	0/0/4	0/2	0/2	this study	Eastern (2)
		**Total**	**0/0/599**	**0/94**	**0/94**		
North Rhine-Westphalia	80	Billerbeck	$5/0/25^{(12)}$	$0/11^{(11)}$	$0/11^{(11)}$	this study, Reil, Rosenfeld et al., unpubl. data and Ettinger et al., 2012	Western (11)
	81	Cologne	$1/0/10^{(10)}$	$2/10^{(10)}$	$2/10^{(10)}$	Rosenfeld et al., unpubl. data, Essbauer et al., 2007	Western (10)
	82	Warburg	0/0/10	0/10	0/10	this study	Western(10)
		**Total**	**6/0/45**	**2/31**	**2/31**		
Rhineland-Palatinate	83	Koblenz				Schilling et al., 2007	n.d.
		**Total**					
Saxony	84	Eisdorf	†	0/1	0/1	this study	Western (1)
	85	Görlitz	0/0/1	n.d.	n.d.	this study	n.d.
	86	Holzhau	†	†	†	this study	Western (1)
	87	Kitzen	†	0/1	0/1	this study	Western (1)
	88	Königshain	0/0/3	0/1	0/1	this study	Western (1)
	89	Leipzig	0/0/11	0/11	0/11	this study	Western (11)
	90	Meschwitz	0/0/1	0/1	0/1	this study	Western (1)
	91	Neukollm	0/0/38	0/1	0/1	this study	Western (1)
	92	Wermsdorf	0/0/2	0/2	0/2	this study	Western (2)
		**Total**	**0/0/56**	**0/18**	**0/18**		
Saxony-Anhalt	93	Calvörde	0/0/9	0/9	0/9	this study	Western (7); Eastern (2)
	94	Golmenglin	0/0/11	0/1	0/1	this study	Western (1)
	95	Heteborn	0/0/12	0/1	0/1	this study	Western (1)
	96	Morl	0/0/20	n.d.	n.d.	this study	n.d.
	97	Walbeck	2/0/40	2/11	0/9	this study	Western (7); Eastern (4)
		**Total**	**2/0/92**	**2/22**	**0/20**		
Schleswig-Holstein	98	Schrevendorf	0/0/22	0/11	0/11	this study	Western (10); Eastern (1)
		**Total**	**0/0/22**	**0/11**	**0/11**		
Thuringia	99	Altenburg	0/0/1	n.d.	n.d.	this study	n.d.
	100	Diedorf	$22/0/58^{(58)}$	$17/22^{(22)}$	n.d.	this study and Faber et al., 2013	Western (16)
	101	Erfurt	0/0/6	n.d.	n.d.	this study	n.d.
	102	Gotha	$0/5/377^{(372)}$	$0/17^{(12)}$	$0/17^{(12)}$	this study and Reil, Rosenfeld et al., unpubl. data	Western (17)
	103	Leutenberg	0/0/2	n.d.	n.d.	this study	n.d.
	104	Lucka	0/0/15	n.d.	n.d.	this study	n.d.
	105	Treben	0/0/4	n.d.	n.d.	this study	n.d.
		**Total**	**22/5/463**	**17/39**	**0/17**		

*If bank voles from other studies were included into the results, the total number of voles added is given within superscript parenthesis. n.d., not determined; PUUV, Puumala virus; RT-PCR, reverse transcription PCR.

†Only few or no samples for ELISA or PUUV RT-PCR were available.

‡No. of positive/ no. of equivocal/ total no. of bank voles tested.

§No. of positive/ total no. of bank voles tested.

Real-time S segment RT-PCR: 2.5 µl RNA template, 20 pmol/µl of primers PUUV S-broad-F, and PUUV S-broad-R, and 5 pmol/µl of the fluorescence reporter probe PUUV S-broad (all purchased from Metabion, München, Germany) were used in a total volume of 12.5 µl with qScript XLT One-Step RT-qPCR ToughMix (Quanta BioSciences, Gaithersburg, ML, USA). The reverse transcription was maintained for 10 min at 50°C followed by 1 min inactivation at 95°C. Amplification of cDNA was performed in 45 cycles, starting with denaturation at 95°C for 10 s, annealing at 57°C for 30 s and elongation with 30 s at 68°C.

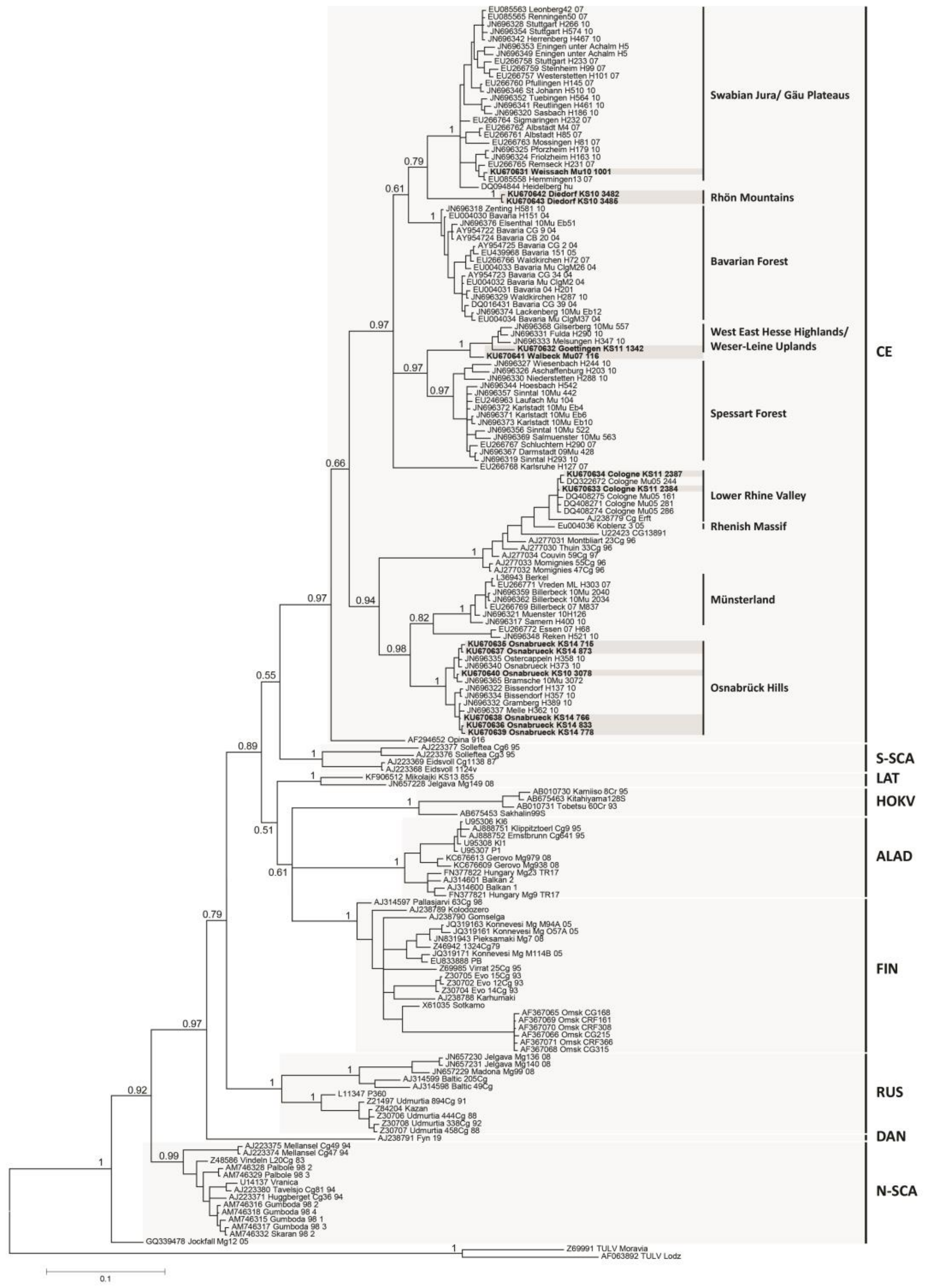

Technical Appendix Figure. Puumala virus (PUUV) phylogenetic tree reconstructed with novel and published partial S segment sequences. Published sequences are labeled with GenBank accession numbers. Novel sequences are given in bold type and are highlighted in dark gray. Posterior probabilities for major nodes of the maximum clade credibility phylogenetic tree are displayed. Phylogenetic analyses were performed with MrBayes version 3.2.2 (https://sourceforge.net/projects/mrbayes/files/mrbayes/) on

the CIPRES platform for 171 PUUV sequences of 465-bp length. A mixed nucleotide substitution matrix was specified in 4 independent runs of 10^7 generations. The data was partitioned into 2 groups: 1) 1st and 2nd codon positions and 2) 3rd codon position. Scale bar indicates nucleotide substitutions per site. A burn-in fraction of 25% was discarded and samples were recorded every 10^3 generations. Tula virus (TULV) was used as an outgroup. ALAD, Alpe-Adrian lineage; CE, Central European lineage; DAN, Danish lineage; FIN, Finnish lineage; HOKV, PUUV-like Hokkaido virus; LAT, Latvian lineage; N-SCA, North-Scandinavian lineage; RUS, Russian lineage; S-SCA, South-Scandinavian lineage.

References

1. Drewes S, Turni H, Rosenfeld UM, Obiegala A, Strakova P, Imholt C, et al. Reservoir-driven heterogeneous distribution of recorded human Puumala virus cases in South-West Germany. Zoonoses Public Health. In press 2016.

2. Essbauer SS, Schmidt-Chanasit J, Madeja EL, Wegener W, Friedrich R, Petraityte R, et al. Nephropathia epidemica in metropolitan area, Germany. Emerg Infect Dis. 2007;13:1271–3. PubMed http://dx.doi.org/10.3201/eid1308.061425

3. Ettinger J, Hofmann J, Enders M, Tewald F, Oehme RM, Rosenfeld UM, et al. Multiple synchronous outbreaks of Puumala virus, Germany, 2010. Emerg Infect Dis. 2012;18:1461–4. PubMed http://dx.doi.org/10.3201/eid1809.111447

4. Faber M, Wollny T, Schlegel M, Wanka KM, Thiel J, Frank C, et al. Puumala virus outbreak in western Thuringia, Germany, 2010: epidemiology and strain identification. Zoonoses Public Health. 2013;60:549–54. PubMed http://dx.doi.org/10.1111/zph.12037

5. Mertens M, Kindler E, Emmerich P, Esser J, Wagner-Wiening C, Wölfel R, et al. Phylogenetic analysis of Puumala virus subtype Bavaria, characterization and diagnostic use of its recombinant nucleocapsid protein. Virus Genes. 2011;43:177–91. PubMed http://dx.doi.org/10.1007/s11262-011-0620-x

6. Morger J, Råberg L, Hille SM, Helsen S, Štefka J, Al-Sabi MM, et al. Distinct haplotype structure at the innate immune receptor toll-like receptor 2 across bank vole populations and lineages in Europe. Biol J Linn Soc Lond. 2015;116:124–33. http://dx.doi.org/10.1111/bij.12593

7. Schilling S, Emmerich P, Klempa B, Auste B, Schnaith E, Schmitz H, et al. Hantavirus disease outbreak in Germany: limitations of routine serological diagnostics and clustering of virus sequences of human and rodent origin. J Clin Microbiol. 2007;45:3008–14. PubMed http://dx.doi.org/10.1128/JCM.02573-06

8. Weber de Melo V, Sheikh Ali H, Freise J, Kühnert D, Essbauer S, Mertens M, et al. Spatiotemporal dynamics of Puumala hantavirus associated with its rodent host, *Myodes glareolus*. Evol Appl. 2015;8:545–59. PubMed http://dx.doi.org/10.1111/eva.12263
